# The Moral Pitfalls of Cultivated meat: Complementing Utilitarian Perspective with eco-republican Justice Approach

**DOI:** 10.1007/s10806-022-09896-1

**Published:** 2022-11-22

**Authors:** Cristian Moyano-Fernández

**Affiliations:** 1 Department of Philosophy, Universitat Autònoma de Barcelona (UAB), Barcelona, Spain; 2 Institute of Philosophy, Spanish National Research Council (CSIC), Madrid, Spain

**Keywords:** Cultivated Meat, Utilitarianism, Sovereignty, Eco-republicanism, Capabilities, Flourishing

## Abstract

The context of accelerated climate change, environmental pollution, ecosystems depletion, loss of biodiversity and growing undernutrition has led human societies to a crossroads where food systems require transformation. New agricultural practices are being advocated in order to achieve food security and face environmental challenges. Cultivated meat has recently been considered one of the most desired alternatives by animal rights advocates because it promises to ensure nutrition for all people while dramatically reducing ecological impacts and animal suffering. It is therefore presented as one of the fairest means of food production for the coming decades, according to utilitarian arguments.

However, food security, environmental concerns and animal welfarism guided by a short-term utilitarianism could have techno-optimism bias and could result in some forms of oppression such as anthropocentrism. I argue that there are still deep-rooted moral issues in food systems that are not addressed primarily by lab-grown meat, mainly derived from a loss of sovereignty. Food practices developed in high-tech labs with artificial interventionism constrain the ability of living entities (that are used as food) to flourish on their own terms. This paper aims to explore how sovereignty entitlements for humans and nonhumans are often overlooked by advocates of cultivated meat and the moral challenges it may pose. Accordingly, a more than utilitarian approach framed by ecological and republican justice is proposed here to shed light on some pitfalls of food chains based on cellular agriculture.

## Introduction

Food and climate change are closely linked. Agricultural and farming processes have the potential to influence the planet’s climate and, in turn, climate affects food production. This feedback has prompted countless researchers to investigate which foods or production methods have the least environmental and climate impact, and how to prevent climate from damaging food production.

Intensive and oil-dependent methods have provided an apparent alternative in light of the Malthusian concern to ensure food for a human population that is growing almost exponentially. By means of techniques such as the use of chemicals, mechanization of processes and irrigation of the land it has been possible to increase the productive capacity of the land by replenishing soil with minerals. However, maintaining this rate of food production to meet the growing demand without causing environmental degradation remains a challenge. The enormous use of water and energy required by intensive livestock farming, for example, is unsustainable in the long term (Pluhar, 2013). Since the second half of the 20th century, intensive livestock farming has profoundly transformed the socioecological metabolisms of many environments and greatly changed our lifestyles and the ecosystem rhythms of the biosphere (Reisinger & Clark, 2018).

In addition to the above, since the recent Covid-19 pandemic there has been a growing concern about zoonotic diseases. This places the spotlight on industrial livestock, who live in conditions of poor hygiene and overcrowding, which can favor the appearance of such mutations and the spread of viruses that are also harmful to our societies (Espinosa et al., 2020). Intensive farming, where the interface between human and nonhuman animals is so narrow, makes viral jumps among species easier.

Given this scenario, we may consider the alternative of cultivated meat, also known as cultured, in vitro, lab-grown, artificial or synthetic meat, because it is presented as the long-awaited solution that will maintain expensive lifestyles without entailing a significant cost to the planet, other sentient beings and our future generations. This raises the question: Why choose such a high-tech process to obtain meat products over other alternatives which have no massive industrial effects?

Despite the survival of some traditional practices like extensive livestock farming, which is dedicated to satisfying people’s taste for meat and strong cultural traditions, it has unresolved underlying problems. On the one hand, the planet does not have enough surface area for extensive livestock rearing to sustainably supply current trends in diets rich in meat proteins (Hayek et al., 2020). Should this practice be chosen as a replacement for intensive livestock farming, there would have to be a drastic reduction in meat consumption. This, in turn, would lead to the other alternative of changing dietary preferences towards vegetarianism or veganism, because either of these would potentially free up around 76% of the land dedicated to agriculture and livestock (Poore & Necemeck, 2018). What is more, extensive livestock farming includes the slaughtering of sentient beings, which poses a serious moral problem from a non-anthropocentric ethical perspective.

What about those people who prefer not to give up meat but are quite reasonably concerned about maintaining good planetary health from an anthropocentric perspective? Many food choices are not logical reasoned actions, but rather automatic decisions relying on heuristic processing and heavily influenced by contextual patterns (Stubbs et al., 2018). Despite knowing the detrimental effects of meat on the biosphere, many people, environmentalists among them, find it difficult to sufficiently reduce their meat consumption due to a defeatist perception of individual responsibility (Scott et al., 2019).

Faced with this complex scenario, where food can be conceived as an essential leverage point for a just transition towards sustainability but at the same time a difficult habit to transform, the option of cultivated meat secures our attention. However, I will argue that the much-vaunted positive consequences of cultivated meat could have some moral pitfalls that should not be overlooked. In particular, I will address short-termism and the sovereignty value for humans and nonhumans.

The research question this paper explores is as follows: what moral challenges may emerge if we rethink cultivated meat from strictly utilitarian perspectives? Cultivated meat is usually defended with arguments centered around its consequences, especially in the short term, and advantages regarding the cost-benefit relationship in comparison to an agribusiness model (Dutkiewick & Abrell, 2021). Although utilitarian reasoning may have moral justification in a quantitative assessment of cultivated meat, it may not address concerns over which qualitative parameters are included within the quantitative balance. That is, it does not sufficiently serve to critically discuss which moral values are being considered and which are not. Furthermore, it is of no help when rethinking who to include in the decision-making process and who to exclude from it, or in questioning the structural hierarchies of power. Thus, we are left to wonder which gaps may be unaddressed by utilitarianism, such as care for sovereignty, and how more-than utilitarian approaches may be helpful in this regard.

It is important to inquire beyond consequence-focused arguments in support of cultivated meat, because producing food is not just about obtaining a more efficient end product, but rather involves a whole set of dynamic and interdependent relationships. And diverse values coexist in these relationships that, in order to be morally respected in a plural and non-dominated way, would need to be embraced by more than utilitarian approaches. This leads me to explore a republican and ecological perspective of justice. Such approaches provide visibility regarding who participates in the decision-making process when it comes to a fairer means of sourcing food in a context of socioecological crisis, who is made invisible, and which moral values cannot by substituted by beneficial outcomes.

The main aim of this paper, then, is to discuss why utilitarian arguments should dialogue with arguments framed from an eco-republican justice to address the moral pitfalls of cultivatedmeat.^1^ It is structured as follows: First, some benefits of cultivated meat will be mentioned, followed by the long-term scalability consequences that need to be considered in a stronger utilitarian defense of this food strategy. In the next section, an eco-republican perspective of justice will be presented, not as an alternative to replace utilitarian arguments for cultivated meat, but to complement the ethical analysis and provide a more in-depth analysis of some issues not sufficiently addressed by consequence-centered lenses. Republicanism raises objections to the structures of domination that result from dependence on high technologies. Ecologism addresses oppression via a critique of the prevailing anthropocentrism in capitalist societies and a concern for the more-than-human world. After presenting eco-republican justice as a helpful approach for constructing normative judgments on cultivated meat, I will specify how it can help detect those scenarios where there is a loss of sovereignty value. Among humans, sovereignty can be lost when opportunities to participate in the food system are reduced. However, there could be more local alternatives that offer community integration in the process of in vitro meat generation. Regarding nonhumans, I will discuss sovereignty more broadly and draw on some contributions from the capabilities approach, reasoning that cultivated meat may cause loss of sovereignty if animals’ capabilities to exercise control over their own bodies and their territorial environment and to relate to other individuals are not respected. And finally, I will discuss how eco-republican justice leads to a dialogue with other normative frameworks and a decolonial attitude in which the cultural acceptance of developing synthetic meat from only some animal species is epistemologically questioned.

## Cultivated meat from a Utilitarian Point of view

Since the first laboratory-created hamburger was presented and tasted in 2013, the funding of research with animal cells to create this synthetic food and public expectations surrounding it have greatly increased (Treist, 2021). Investigating artificial meat is neither technologically simple nor cheap, the high cost being a factor that slows down the process (Rubio et al., 2020; Stephens et al., 2018). However, proponents of cultivated meat expect production to become cheaper if the consumption of artificial meat becomes widespread or private, philanthropic, and public sector investors decide to put more money into it.

It is often argued that cultivated meat production would significantly alleviate some of the major environmental problems associated with intensive livestock farming, such as animal suffering, overexploitation of land and water, methane emissions, deforestation, and fertilizer, pesticide, and fossil fuel abuse (Rubio, et al. 2020; Post, 2012). In theory, it presents advantages that minimize injustices towards animals, the environment and people, meaning it has many benefits from a utilitarian point of view (Pluhar, 2010; Hopkins & Dacey, 2008).

With cellular farming, far less animal slaughter would be required, or even none at all, to manufacture meat products compared to industrial livestock farming. Furthermore, its processes of technological intensification in cell culture laboratories would entail a “land sparing” strategy -as it is usually called within the field of conservation biology (Mertz & Mertens, 2017)- by means of which much more land would be released than is currently maintained for livestock. This would have the benefits of leaving more natural surface for wildlife, which constitutes another compelling reason in its favor for those concerned about animal suffering (Schaefer & Savulescu, 2014).

Cultivated meat also promises to address environmental challenges by conserving land and water, preserving habitat, reducing greenhouse gas emissions, and preventing manure pollution and antibiotic overuse (Post, 2012; Tuomisto & Teixeira de Mattos, 2011). Proponents of cultivated meat have suggested that it generates lower emissions per unit of meat produced than current livestock production, and even fewer if renewable sources are used to run bioreactors and ensure global scalability of the product (Simon Nobre, 2022).

In addition, cultivated meat offers a solution to human population growth and the trend of increasingly preferring an animal-based diet (Post & Hocquette, 2017). It is expected that the artificial reproduction of cells will generate surplus protein food for a population of 10 billion people, while requiring fewer resources and animals than current industrial livestock farming (Bryant & Barnett, 2020; Post & Hocquette, 2017).

In sum, then, the watchwords for this transition to cellular farming are that it would increase animal welfare, reduce environmental costs and sustain human health by providing food for the world’s population. All these potential benefits are often considered sufficient to at least support the cultivated meat process from a consequentialist point of view. However, despite the possibility that it could be produced on a large scale, there would still be epistemological and ethical problems. There are some flaws in the utilitarian approach used to support cultivated meat, some of which can even be discussed from a consequentialist point of view. The key issue based on utilitarian arguments is to weigh how much suffering is saved by generating cultivated meat (Dutkiewick & Abrell, 2021). And here the answer basically depends on three main conditions: the time scale used to calculate the trade-offs; what we understand by suffering; and who is recognized as having the basic capacity for suffering.

The second and the third variables appeal to more than utilitarian approaches, which is why I will take them up in the latter sections after presenting the eco-republican perspective. But the first, concerning timescale, relies on consequentialist analysis and is intertwined with different nuances of utilitarianism. I therefore mention timescale here as a potential determining factor in understanding the environmental consequences that the defense of cultivated meat based on utilitarian arguments might face. The epistemological methodology used to defend the beneficial outcomes of cellular agriculture may come up against some challenges when calculating energy savings and environmental impacts.

To the end discussed above, it would be appropriate to compare at least two temporal scenarios, short-term and long-term, given that the resulting balance may well differ to some extent. Regarding the supposed environmental benefits of cultivated meat, it must be considered to have less environmental impact than beef and perhaps pork, but more than chicken and plant-based proteins (Lynch & Pierrehumbert, 2019; Smetana et al., 2015). While the technology used in the process has significant scope for innovation that could reduce energy requirements below these assessments and offer better environmental results in the long-term (Stephens et al., 2018), it cannot currently be deemed an environmentally better solution than directly advocating for a reduction in meat consumption. In fact, cultivated meat still faces challenges such as using low-cost non-animal growth cultures and designing bioreactors (Tuomisto, 2019).

In addition, the Jevons paradox should be considered, which posits that stimulating technological efficiency in the use of resources can lead to their greater consumption, thereby canceling out energy and environmental savings (Polimeni et al., 2009). In other words, expanding production of cultivated meat could have a “rebound effect”, in which total energy consumption would grow due to increased demand resulting from the existence of cheaper products and greater efficiency during the process. If the promotion of synthetic meat proves convincing to the public and succeeds in the marketplace thanks to rhetorical strategies, this may lead to fewer people opting for plant-based foods (even if they are more sustainable). Although cultivated meat could reduce the overall amount of animal suffering on a short time scale, on a longer one it could perhaps hit a ceiling, at which point it could no longer be significantly reduced. Lately, the perspective of long-termism has been included within utilitarianism, so a consequentialist defense of cultivated meat should also consider it.

## Eco-republicanism to Confront the Potential Oppression of Cultivated meat

Even if cultivated meat proponents embrace long-termism, arguing utilitarian reasons, they still focus on and prioritize outcomes over processes, and some moral-based assumptions are perpetuated without necessarily being rethought. The questions posed earlier were: what do we understand by suffering, and who is recognized as having the basic capacity for suffering? Relying solely on utilitarian criteria to defend cultivated meat would not be sufficient to properly address some moral pitfalls deriving from such questions.

All food chains entail several profound issues that need to be dealt with. I argue that, in addition to criticisms of cultivated meat that may come from the long-term utilitarian perspective, there are other controversial points that deserve to be addressed from a republican and ecological justice approach. Power and domination should also be studied to determine which collectives are privileged and which remain under dependency through cultivated meat production, including human and nonhuman animals in this moral inquiry. Two approaches can be considered in respect of this: the republican and the ecological.

### The Republican Perspective Preventing Social Oppression by the high-tech World

The utilitarian perspective of food security in the Anthropocene context (Noll, 2019) considers the advantages resulting from calculating the large amount of food that can be produced by cultivated meat thanks to reduced energy and environmental costs. Food production, resource consumption and certain environmental impacts are more or less measurable. The distribution of products, and more specifically in our case food, that would accompany utilitarian philosophy focuses on the collectivist metric of achieving the greatest good for the greatest number of individuals (Bentham, 1996 [1789]). In this case, the sacrifice of individual rights for the public good, that is, increased food production, could be justified. However, the resulting utilitarian balance of blindly prioritizing food supplies over the respecting of certain inviolable values and rights could unjustly perpetuate oppressive relationships.

The republican perspective goes beyond concerns about quantitative distributions mediated by utilitarianism, because it is more sensitive to power and domination relations (Pettit, 1997). The republican position considers participation in the public sphere to be a constitutive element of citizenship (Lozano-Cabedo & Gómez-Benito, 2017). Thus, republicanism is committed to determining which collectives are privileged and which remain under dependency through cultivated meat production and distribution. Cultivated meat is a product from the high-tech world, and restricting production to this technology alone perpetuates the exclusion of key players aiming to create a sustainable food future. Farmers and land sovereigntists should be important players in our food systems (Borras et al., 2015), since they have strong local and traditional knowledge of the land (Engdawork & Bork, 2015) and of how to grow food while keeping the soil healthy (Rhodes, 2012). Nevertheless, shifting food systems to cultivated meat and high-tech processes could aggravate the loss of traditions based on respectful interactions with ecosystems and experience in obtaining food in a sustainable and regenerative way.

Some authors have pointed out that the focus on technological solutions to food security unfortunately “minimizes the need for difficult ethical reflection on our industrialized way of life in relation to either the poor or to the natural environment” (Rush, 2013). Industrializing systems and enhancing the reliance on technologies often cause further damage to rural collectives and people who decide to live without so many technological dependencies, or who cannot afford to live according to this expensive system of machines, devices and ultra-processed products. In this developed model, cultivated meat might contribute to marginalizing some collectives and make them more vulnerable.

While there is still domination among nations, communities and species, and power distribution is the hands of a small group of individuals, externalities will remain invisible and there will be no empowerment or food sovereignty. Not everyone is able to develop cellular agriculture, because prior specific knowledge of biotechnology, laboratories full of machinery, competent teams and industrialized systems are all required to this end. Consequently, the conditions to produce and obtain food would be extremely limited to a privileged sector of society and distributive procedures would broadly depend on this. The technological, technical and economic restriction to participate in the in vitro processes of food generation is far from a real democratization of food systems and close to a discrimination that further disconnects people from the land, nonhuman animals and self-sufficiency.

### The Ecological Perspective Averting Anthropocentric Domination

The loss of food sovereignty and the different forms of social oppression that could arise as an unforeseen consequence of cultivated meat is a republican critique that requires further examination if we are to evaluate the moral validity of developing and producing this foodstuff. The main contribution of ecological justice is aligned with the republican concern over injustices based on domination: applying the concept of justice to the nonhuman world (Dobson, 2006). Although utilitarianism may be a helpful perspective in applied ethics and justice, it may also be based on moral ontologies that need to be rethought. The ecological perspective shines a light on the weakening and even fragmentation of anthropocentric ontologies by including the nonhuman world within the moral scope (Donoso, 2020).

Regarding concerns over food justice, an ecological view aims to recognize which more-than-human entities are being instrumentalized and severely exploited in the food production chain. Distributive theories of ecological justice broaden the spectrum of beings considered as beneficiaries of the fair distribution of resources (Baxter, 2005). Recognition and participatory theories of ecological justice delve deeper and ask how beings and communities should be included in justice and how humans might listen to them without imposing our own voice (Dryzek, 1995). From this perspective, it is often assumed that nonhuman nature and even ecosystems have a certain agency worthy of mention. The use of agency as a morally relevant characteristic is somewhat more problematic, however, because its scope can be very narrow if it is linked to ideas such as cognitive intentionality or self-awareness.

That being said, agency does not imply assuming a superstitious personification regarding nonhuman nature: even if it lacks the capability for rationality, it could hold moral rights if classic reciprocity between rights and duties is abandoned (Curry, 2000). Many animals and ecosystems do not express themselves via cognitive strategies or make claims based on reasons like humans. They evolve in line with the laws of physics, chemical reactions and biological interactions. Rationality or sentience are not a necessary condition for an entity to become a victim of injustice, but other capacities, such as resilience, can be measured to discover how some entities change patterns when under stress conditions (Kortetmäki, 2017).

The central issue in evaluating whether cultivated meat is permissible from an ecological perspective essentially boils down to a question of whether nonhuman entities form part of food decision-making and, if so, in what way. This requires learning how to listen to other voices in food regeneration processes and include their autonomy or preferences. Thus, the ecological perspective embraces caring for nonhuman entities involved in food chains and trying to represent their own sovereignty. In accordance with this approach, rather than an end product, food may be considered an integral part of an interdependent chain. This suggests that cultivated meat should be analyzed by considering how humans respect nonhuman nature during the food generation process and ensuring that their treatment does not slip into forms of animal disenhancement (Thompson, 2020: 355–358).

## Preserving the Value of Sovereignty at Human and Nonhuman Scale

Both perspectives, republican and ecological, have a common concern for the value of sovereignty. The former mainly addresses human sovereignty (Lozano-Cabedo & Gómez-Benito, 2017) and the latter nonhuman sovereignty (Donoso, 2020). Despite both being presented separately, together they may help rethink those interfaces where human and nonhuman capabilities are intertwined in a world partially shaped by food.

Consequentialist analyses focus mainly on how much collective well-being is gained through the procedure of producing cultivated meat, assessing the extent to which it is worth promoting this food transition, even if it implies accepting some trade-offs. The key argument of the thesis advocating for the utilitarian advantages of cultivated meat is that, from a basically quantitative approach, the benefits outweigh the costs.

However, I oppose the view that numerical calculations deriving from the cost-benefit mantra will suffice to morally evaluate cultivated meat. Cultivated meat has some moral pitfalls in addition to the beneficial consequences it may generate. The logic that leads utilitarianism to measure the overall calculation can overshadow the particularities of each form of life involved in the process of generating and receiving food. There are values whose loss cannot be offset by the gain of other values. These are interchangeable minimums of justice.

Specifically, here I address the value of sovereignty in relation to two types of sentient beings: human and nonhuman animals. It is worth noting that I understand sovereignty in a broad sense, as freedom, autonomy, or within the Senian meaning of capability: having the opportunities to be or do what one finds valuable in order to flourish (Sen, 1999). From this perspective, freedom of choice or sovereignty has not only an instrumental value (it is valuable as a means to an end), but also an intrinsic one; that is, it is valuable in itself, for the well-being of an individual. The ability to decide how one prefers to flourish is directly related to an individual’s quality of life. And if sovereignty is not respected, then slow and silent suffering may ensue. This need not necessarily be associated with bodily pain, as it can also be cognitive or derive from a limitation in basic capabilities to flourish.

Food strategies aimed at addressing climate challenges should not result in a loss of sovereignty, which would be a process linked to suffering (Noll & Murdock, 2020). Any proposed eco-authoritarian alternative should be reviewed and discussed, as it is detrimental to the ability to flourish according to each individual’s own conception of a good life.

Faced with the current socioecological crisis characterizing the Anthropocene and the increasingly demonstrated correlations between animal-protein diets based on industrialization and aggravation of this crisis (Reisinger & Clark, 2018), a growing number of people advocate for renouncing some of the more democratic ideals and undertaking more autarkic measures. Thus, although ecoauthoritarianism remains a marginal political movement, it is gaining adherents as the global environmental context worsens (Man & Mainwright, 2017). Is this fair though? What about the sovereignty of the subjects concerned?

Here, in discussing the validity of a fully ecoauthoritarian system that would impose a sort of green Leviathan, I am not rejecting the idea of a hierarchy in the decision-making process with regard to food systems. What I am criticizing is the fact that applying utilitarian criteria to efficiently obtain outcomes advantageous to the majority may end up being the only approaches considered, given their weak commitment to inter-individual differences. It is important to focus our critique on the number of individuals participating in the political process that makes decisions in a quantitative sense about, for example, food. But it is also crucial to discern who are the most involved and influential subjects and who, on the other hand, are being left invisible and ignored.

### Claiming food Sovereignty for Humans

Accelerated climate change affects the capabilities and sovereignty of both human and nonhuman beings. But food practices developed in response to the environmental crisis also affect freedom in a variety of ways.

First, I will focus on the loss of sovereignty for human beings. In the debate on how to protect sustainable human development, it seems that many discourses tend to point primarily towards the protection of food security. Having food is a basic right, because it is a necessary condition for being able to flourish with dignity. Without being well nourished and healthy, one will hardly be able to decide how one desires to be or continue to function. Hence, it is reasonable to aim to ensure that we all have food. But it is also reasonable to be concerned about how we can obtain that food, and about who is included in the food production and supply systems. That is, to attend to sovereignty from a human-centered morality.

One aspect of consumer sovereignty is voluntariness (Mepham, 1996), but to ensure voluntariness in the choice of food can at times be complicated. When a consumer buys a food product, this does not imply that there is a consent to buy exactly that product. Consumer preferences may be conditioned by the options available at the grocery store, for instance (Röcklinsberg, 2006). Thus, a republican perspective would propose having the sovereignty to decide no longer what we *want* to buy but what we *can* buy. Having the choice to be part of the decision-making process about which food is sold, at what price and under which conditions, should be a legitimate claim to justice (Höglund, 2020).

Losing the right to food sovereignty implies losing a value that cannot be compensated for by gaining other benefits, such as receiving certain resources or food products (Noll & Murdock, 2020). On the one hand, we should be able to know how food is made throughout its production chain. This would imply having a more in-depth understanding of our relationship with the nonhuman animals used in this process, as well as the resulting impacts on the land and environment. On the other hand, we should at least have the option to participate in food-making processes in order to be self-sufficient. I think that the recent Covid-19 pandemic has awakened a legitimate interest and concern in many people to become more resilient and not to depend on transporters and supermarkets to be able to have enough food. Hence, a good number of people have begun to set up small gardens and grow their own food in the gardens and balconies of their homes (Sofo & Sofo, 2020).

Seeking this food autonomy and recognizing the interdependencies generated during food production are not currently targets contemplated in the transition to cultivated meat. Some authors have suggested a hypothetical scenario known as the “pig in the backyard” as an alternative to preserve food sovereignty and self-sufficiency during the production of cultivated meat (Van der Weele & Driessen, 2019). According to these authors, we should consider the possibility of cultivated meat being produced by cell extraction from a pig in the backyard of our homes or our communities. This possibility would defeat objections that in vitro meat is neither local nor conducive to food sovereignty. However, at present, with large biotechnology companies leading the way in cultivated meat production, this still seems a distant scenario. In addition, even if this process were to ensure sovereignty for humans, it remains to be seen how it might ensure sovereignty for nonhumans.

### Claiming Sovereignty for Nonhumans

In the case of humans, I have focused on addressing the value of sovereignty in relation to what I have called the food concern, understanding this as the freedom to produce and manage one’s own food, because I believe that there are already sufficient arguments for sovereignty to be considered an inviolable value. For nonhumans, however, I will not discuss sovereignty in relation to food so much, as I will approach it from a broader consideration. I will appeal, above all, to their capabilities to exercise control over their own bodies -to have bodily integrity-, to exercise control over their surrounding environment, and to relate to other members of their own and other species (Nussbaum, 2006).

In those animals used to produce synthetic food, the biopsy that accompanies the process of cultivated meat generation certainly need not cause any apparently direct disrespect for the animal’s sovereignty. The extraction of cellular tissue is painless and does not have to damage its physical integrity. But what about the culture medium? Cultivated meat requires fetal bovine serum for food growth in the laboratory, a culture based on calf stem cells. After a mother cow has been slaughtered and gutted, her uterus, which contains the fetus, is removed. Only fetuses older than three months are used, otherwise the heart is too small to perforate (Lanzoni et al., 2022).

Although there are already tests with other culture media that do not require animal embryonic cells, such as algae, the most widely adopted option which offers the best results remains the use of mammalian fetuses (Hocquette, 2016). It is here that the variable of which subjects are recognized as capable of suffering and thus deserving of moral consideration comes into play, establishing a dialogue with the question of what is understood by suffering. If the fetuses used during in vitro meat processing are understood as sentient beings, with intrinsic value and with a whole life ahead of them to potentially flourish, then the cost-benefit scale of cultivated meat may not be as advantageous as often presumed. Here, the prior adjudication of moral status to some individuals or entities -or not- may influence the utilitarian advantages that can be appreciated in cultivated meat. Therefore, further interdisciplinary research on animal minds and sentience is required in order to dig deeper into the moral assessment of this variable conditioning the cost-benefits of cellular agriculture.

Returning to the ethical analysis of the biopsy process, there are yet other challenges that need to be addressed in order to preserve sovereignty. Depending on the location of the biopsied animals, there may be potential damage to their ability to develop freely in an environment and in relation to their fellows and other species. If, for example, in order to facilitate and accelerate cell extraction, the animals were forced to be in a reduced space and in conditions of overcrowding, then a moral problem would arise, as their territorial autonomy and freedom to interact with other individuals would not be respected. As Donaldson and Kymlicka pointed out (2011), animals should have the political right to enjoy the sovereignty of their communities and territories.

Even so, we could imagine an extended version of the hypothetical “pig in the backyard” proposal and assume that the animals from which we would extract the cells would be placed in large community yards, such as some animal sanctuaries or reserves, where they would live in coexistence with other species and could have ample freedom of movement. Preventing the biopsy from causing harm to the animal’s basic capabilities would seem to avoid the moral pitfall that cultivated meat might cause from a non-anthropocentric perspective.

All that being said, as mentioned above, the production of cultivated meat also requires fetal bovine serum for food growth in the laboratory, a culture generally based on millions of calf stem cells (Hocquette, 2016). This poses another ethical challenge. Beyond moral discussions regarding the possible intrinsic value of the bovine fetus, it is worth asking to what extent an animal, generally a cow, from which stem cells are extracted to be used as a culture medium to produce in vitro meat, is not suffering an aggression against its physical integrity. For example, some deontological ethics (Francione & Garner, 2010) would argue that insofar as the cow fertilized to extract stem cells from it is a subject capable of experiencing its own life, it has intrinsic value and, therefore, deserves rights that would hardly grant this non-consensual violation of its body. In focusing on the balance of results that generate less suffering, utilitarianism is not so sensitive to respecting those moral parameters that should be inviolable. In other words, everything can be sacrificed if it generates the greatest benefit for the greatest number of individuals. As we can see here, however, from a deontological or capabilitarian ethics, this mechanism of moral deliberation is not sufficient to address some of the pitfalls of cultivated meat. A dialogue with other ethical perspectives is needed, which integrates a scheme of values that cannot be reduced to quantitative metrics (Dutkiewick & Abrell, 2021). And although cultivated meat is not necessarily a utilitarian practice, its defense may be guided by utilitarian arguments and motivations; it is therefore important to be critical and watchful over the challenges that might arise.

### Critical Distance when Rethinking the Sovereignty Value

It should be noted that cultivated meat does not lead to a loss of absolute sovereignty for nonhuman animals. Actually, it would help to decolonize a large part of the systematic exploitation of the meat industry, guaranteeing respect for many of the basic capabilities of millions of them. In addition, whether a method of intensification or a land sparing strategy, it would allow the liberation of vast natural areas for many species to reappropriate their territories, which is fundamental for respecting one’s own sovereignty and facilitates options for good flourishing. In comparison with the mainstream meat industry, cultivated meat would therefore help regain a fair amount of sovereignty for nonhuman communities. Nonetheless, in contrast and in relation to a hypothetical scenario in which we would all be vegetarians or vegans -producing our plant-based proteins through ecological agriculture- it may be worth asking whether cultivated meat would not be generating losses in the sovereignty of nonhuman animals. This comparison of scenarios may deserve further discussion (Santo et al., 2020).

The above being said, there are some closing remarks that I consider important to bear in mind when criticizing the narrowness of a solely consequentialist defense of cultivated meat from an eco-republican perspective. In previous sections, I have presented the two approaches of republicanism and ecological as being separate, even if I do call for the need to turn to both when morally analyzing cellular agriculture and, in particular, regarding the value of sovereignty. But cultivated meat raises challenges that should be addressed jointly from an eco-republicanism standpoint, since moral concerns from both frameworks are intertwined.

This can be illustrated, for instance, by examining what we decide to be food or not. Such a decision affects both human and non-human sovereignty. According to republicanism, the freedoms of different societies and communities should be equally respected in deciding which animal can be chosen to reproduce its meat in vitro. From the ecological justice point of view, animals’ capabilities to flourish with dignity should be equally respected.

Therefore, the eco-republican perspective is concerned with both cultural diversity and thoughtless anthropocentric exploitation. The tandem of republican and ecological justice could introduce a new lense with which to rethink moral evaluations of cultivated meat focused only on its consequences, whether short- or long-term.

Merely tackling global harms and the reduction of negative consequences -to nonhuman animals, the environment and human beings- entails a moral dissonance when some animal species commonly exploited as food in Western countries -like cows, pigs, chickens and fish- are used to produce cultivated meat, and there is a rejection of using other animals not normally eaten in the culture such as cats, dogs or rats. This has already been discussed in some other studies (Bryant et al., 2019). If it is accepted that cultivated meat be produced from sentient animals like cows and pigs, there should in principle be no moral reasons, other than speciesist prejudices, to exclude the use also of cultivated meat from dogs and other animals. The “non-normalness” of the latter for some societies could raise issues over epistemological colonialism about how we understand cultivated meat on a global scale. This suggests that, while debates about cultivated meat from “unusual” species, and even “ethical cannibalism” (Milburn, 2016) are philosophically interesting, products from non-traditional meat species in Western countries are unlikely to find a large consumer base (Bryant & Barnett, 2020) and are therefore of little practical relevance.

Why may this be relevant from a capabilitiarian and eco-republican justice applied to cultivated meat? Because it shows that, for food ethics, the balance in consequences -between the “bad” and the “good”- is not always a quantitative assessment of trade-offs. Sometimes food ethics requires rethinking which consequences may be morally accepted, which are less permissible, and which are unjustified. This leads to plural and qualitative assessments of what values like sovereignty mean and for whom. The wide range of conceptions each society holds regarding animals reflects how cultural plurality can condition the fairness and global acceptance of cultivated meat, so that non-dominant perspectives must be considered in order to avoid some potential epistemological biases and to not perpetuate colonial preferences.

## Conclusion

It can be difficult to imagine a scenario in which industrial meat based on intensive livestock farming is completely left behind and human nutrition requirements are still completely covered in a changing climate context. But cultivated meat seems to represent a move in this direction. It may have numerous beneficial consequences in the short term, such as causing less animal suffering, reducing some environmental impacts and ensuring food security, but it still holds some moral pitfalls. The cost-benefits from a utilitarian point of view and consequentialist justice need to be discussed in greater depth. Utilitarian approaches focused on analyzing cultivated meat should consider the long-term consequences, for example.

Furthermore, although cultivated meat may bring more benefits than costs compared to the current agribusiness models, there are still some missing values which should be taken into consideration, like sovereignty. Here, the objections provided from a theory of justice based on eco-republicanism are significant. Domination and power relations are not properly addressed by the cultivated meat agenda, and yet they become crucial in an ethical assessment of food systems. Preserving food sovereignty, participatory processes and recognizing the integrity of beings and environments affected by food production should be ethical targets addressed by proponents of cultivated meat.

It is true that cultivated meat may contribute in a high degree to ensuring food security, and even promise better consequences for respecting sovereignty compared to the current agro-industry system. But I consider it ethically important to rethink what we mean by sovereignty and for whom it should be recognized. Strictly utilitarian arguments are rarely concerned with this. In contrast, an eco-republican framework is more likely to question how and why this background has been shaped, since one of its purposes is to embrace thought diversity and avoid domination patterns. As an approach it is related to decolonial epistemologies when it comes to choosing which animals we may use to reproduce their flesh in vitro, without resulting in the unjust oppression of other cultural criteria and other nonhuman beings.

## Footnotes

1 A shorter and earlier version of this paper was published online (Moyano-Fern?ndez, 2021).
